# Genome-wide association study of positive and negative affect reveals shared genetic architecture and a potential causal relationship with cognition

**DOI:** 10.1038/s41598-026-51734-1

**Published:** 2026-05-25

**Authors:** Chloe Slaney, Naoise Mac Giollabhui, Peter J. van der Most, Ensor R. Palacios, Lifelines Cohort Study, Raul Aguirre-Gamboa, Patrick Deelen, Lude Franke, Jan A. Kuivenhoven, Esteban A. Lopera-Maya, Ilja M. Nolte, Serena Sanna, Harold Snieder, Morris A. Swertz, Peter M. Visscher, Judith M. Vonk, Cisca Wijmenga, Naomi Wray, Harold Snieder, Michel Nivard, Gibran Hemani, Catharina A. Hartman, Golam M. Khandaker

**Affiliations:** 1 Medical Research Council Integrative Epidemiology Unit, Bristol Medical School, University of Bristol, Oakfield House, Oakfield Grove, Bristol, BS8 2BN UK; 2 Centre for Academic Mental Health, Population Health Sciences, Bristol Medical School, University of Bristol, Bristol, UK; 3 Depression Clinical and Research Program, Department of Psychiatry, Massachusetts General Hospital, Boston, USA; 4 Department of Epidemiology, University of Groningen, University Medical Center Groningen, Groningen, The Netherlands; 5 Interdisciplinary Center Psychopathology and Emotion Regulation, Department of Psychiatry, University of Groningen, University Medical Center Groningen, Groningen, The Netherlands; 6 NIHR Bristol Biomedical Research Centre and NIHR Bristol Clinical Research Facility, University Hospitals Bristol and Weston NHS Foundation Trust, Bristol, UK, Bristol, UK; 7 Avon and Wiltshire Mental Health Partnership NHS Trust, Bristol, UK; 8 Department of Genetics, University of Groningen, University Medical Center Groningen, Groningen, The Netherlands; 9 Department of Pediatrics, University of Groningen, University Medical Center Groningen, Groningen, The Netherlands; 10 Institute for Molecular Bioscience, The University of Queensland, Brisbane, Queensland Australia

**Keywords:** Diseases, Genetics, Neuroscience, Psychology, Psychology, Risk factors

## Abstract

Altered affect and cognitive dysfunction are burdensome features of many neuropsychiatric conditions that are highly comorbid, remain poorly understood, and have few efficacious treatments. Exploring their genetic architecture and causal relationships may provide insight into their aetiology and comorbidity. Compared to related but distinct traits (depression, wellbeing, neuroticism), findings from genome-wide association studies (GWAS) of positive and negative affect may be informative due to these phenotypes being less heterogenous (compared to broader phenotypes of wellbeing and depression), whilst still retaining important clinical relevance as potential targets for indirect intervention (compared to neuroticism which may be less modifiable). Using data from the Lifelines Cohort Study, we conducted the first GWAS of positive and negative affect using a validated measure (N = 57,946), and four cognitive domains: working memory, reaction time, learning and memory, and executive function (N ≥ 35,729). We then assessed genetic overlap and potential causal relationships using genetic correlation and bidirectional Mendelian randomization (MR) analyses, incorporating large GWAS on related—albeit distinct—phenotypes (depression, anxiety, wellbeing, general cognitive ability [GCA]). We identified one SNP that reached genome-wide significance (*p* < 5 × 10^–8^) for reaction time, and many independent SNPs with suggestive associations for other phenotypes (N = 11–20). For most phenotypes, exploratory gene mapping indicated that SNPs with suggestive associations have higher gene expression in brain tissue compared to other tissues; however, this only met Bonferroni-corrected *p*-value threshold criteria for positive affect and visual learning and memory. Genetic correlations between negative and positive affect suggest that they are dissociable constructs (*r*_g_ = − 0.18). GCA has higher genetic overlap with negative affect than with positive affect (*r*_g_ = − 0.19 vs − 0.06), which could indicate that negative affect and GCA have a higher shared neural basis and/or they exhibit causal relationships. Supporting the latter, MR analyses indicated that higher GCA may reduce negative affect, depression, and anxiety, and increase wellbeing, with little impact on positive affect. Conversely, MR analyses indicated that higher risk of depression and lower wellbeing may causally reduce GCA. Together, these findings suggest that interventions that indirectly influence GCA may be valid targets to prevent negative affect, while interventions that indirectly influence depression/wellbeing may be valid targets for GCA.

**Supplementary Information:**

The online version contains supplementary material available at 10.1038/s41598-026-51734-1.

## Introduction

Positive and negative affect reflect the extent to which a person feels positive or negative emotions (e.g., excited, interested; nervous, distressed), respectively. Positive and negative affect can be experienced in a state (temporary) or trait (stable) manner^1^ and are distinct transdiagnostic features of many health conditions, particularly depression and anxiety. Another critical yet often overlooked transdiagnostic feature of many health conditions is cognitive dysfunction^2,3^. Despite altered affect and cognitive dysfunction being burdensome and pervasive features of many conditions, they remain poorly understood and have few efficacious treatments^2,4^.

Understanding the genetic architecture of mental health and cognitive phenotypes, and their relation to each other, can provide aetiological and biological insights which may facilitate identification of novel treatment targets^5,6^. Although observational studies have found associations between mental health and cognition^8–11^ (although see^7^), causality remains unclear^12^. Reported associations may reflect: (i) poorer mental health causing poorer cognition, (ii) poorer cognition causing poorer mental health, (iii) confounding via shared risk factors (e.g., stress, sedentary behaviour)^13^. Crucially, studies investigating causal relationships between more narrowly defined mental health phenotypes (e.g., affect) and cognitive domains (e.g., memory, attention) are needed^14^. Evidence of bidirectional causality would strengthen the case that interventions indirectly targeting poor mental health (either diagnostic conditions or transdiagnostic phenotypes) may prevent and treat cognitive dysfunction, and vice versa. Two approaches that can shed light on the aetiology, comorbidity, and/or causal relationships between phenotypes are genetic correlations (using all genome-wide variants)^15,16^ and Mendelian randomization (MR) (typically using variants that reach genome-wide significance threshold)^17^. Recent genetic correlation studies highlight the substantial pleiotropic genetic architecture of various cognitive phenotypes with mental health traits^18–20^. Hagenaars et al.^18^ reported large genetic overlap between cognitive measures in UK Biobank (verbal-numerical reasoning, reaction time, memory) and mental health conditions including, but not limited to, major depressive disorder. However, it is unclear why the genetic overlap arises. Some possible explanations include genetic variants impacting traits via shared heritable risk factors (e.g., same neural circuitry), potential causal relationships between traits (i.e., genetic variants impacting cognition which then impacts mental health), or other factors (e.g., assortative mating, linkage disequilibrium). MR can test for causality given certain assumptions are met (see Supplementary Methods). It does this by using genetic variants robustly associated with the exposure as a proxy for it. The properties of genetic variants (random assignment from parents, fixed at conception) mean that they are less likely to be associated with confounders and overcome issues of reverse causality^17,21^. These methods provide powerful tools given the availability of large genome-wide association studies (GWAS) on well-defined phenotypes.

To date, GWAS of mental health phenotypes have largely been conducted on diagnostic categories such as depression and anxiety^5^. However, these conditions are heterogeneous, with diagnosed individuals showing diverse symptom profiles. For example, a diagnosis of depression requires ≥ 5/9 *diverse* symptoms to be present within a two-week period, one of which must be low mood or anhedonia^22^. In addition, symptoms are often not specific to a condition, meaning that a GWAS of one condition may in reality capture a broader phenotype. One potential approach to reducing phenotypic heterogeneity, while addressing the overlap with other conditions, is to focus on *more narrowly defined* traits that more closely map onto biological systems (positive and negative affect, specific cognitive domains)^23^. In line with the Research Domain Criteria framework^24,25^, these are often transdiagnostic.

In this study, we first conducted GWAS of negative and positive affect (assessed using the Positive and Negative Affect Schedule [PANAS]) and performance on cognitive tasks designed to tap into several domains (executive function, working memory, visual learning and memory, and reaction time). Second, to gain greater insight into associated genetic variants, we conducted gene mapping and tissue expression analysis. Third, we investigated genetic overlap between mental health (negative affect, positive affect, depression, anxiety, wellbeing) and cognitive (general cognitive ability [GCA], specific cognitive domains) phenotypes using genetic correlations. Fourth, we tested evidence of potential causality between mental health and cognitive phenotypes using MR analyses. This is the first GWAS conducted on positive and negative affect using well-validated measures of these phenotypes. To achieve this, we use a large Dutch population-based cohort, the Lifelines Cohort. The application of MR to test evidence of causal relationships between affect and cognition is critical for improving our conceptual understanding of the high comorbidity between these phenotypes.

**Fig. 1:**
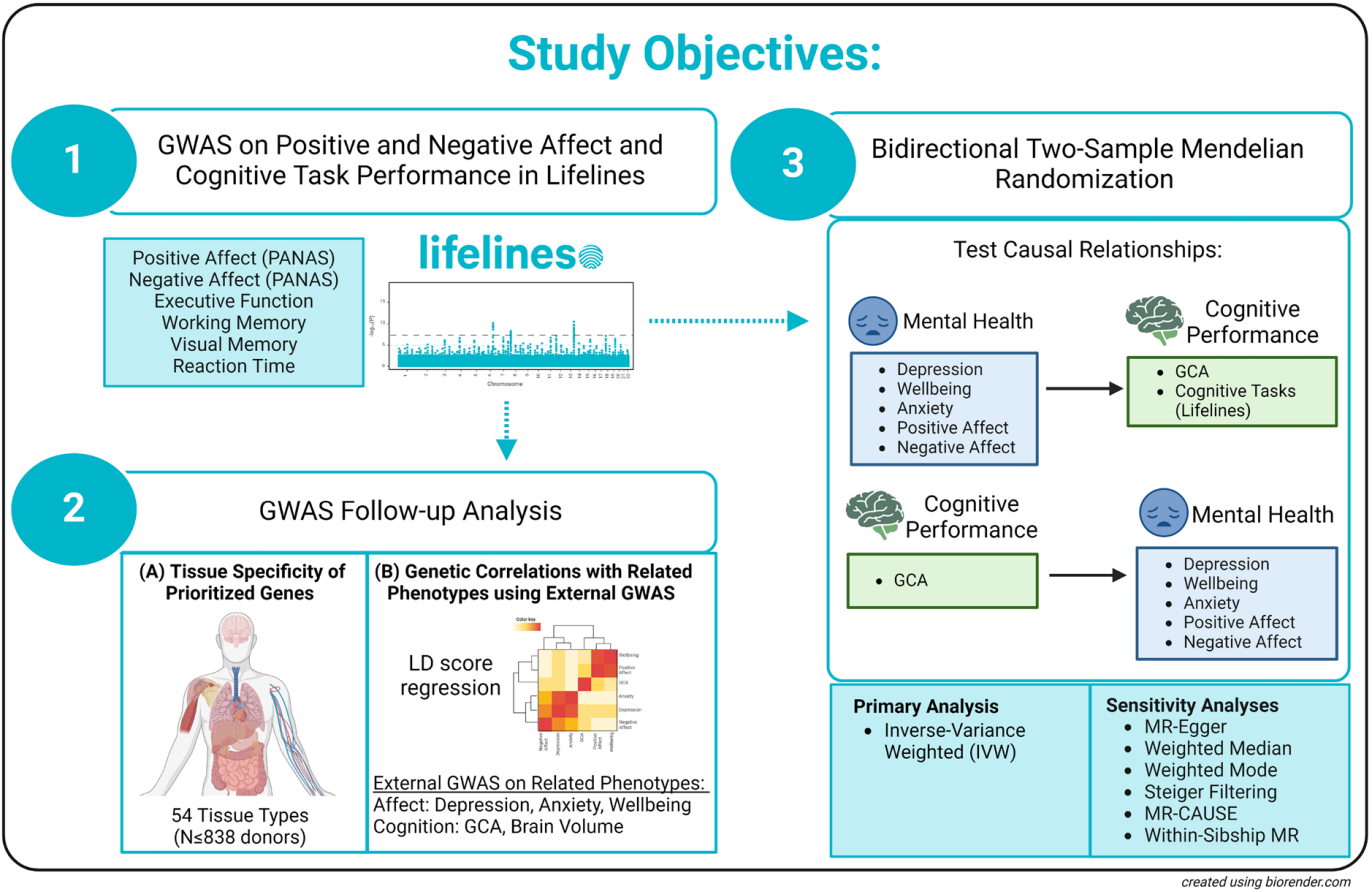
Study objectives.

We hypothesised that: (i) follow-up analyses on all GWAS will show increased gene expression in brain tissue compared to other tissues; (ii) affect and cognitive measures in the Lifelines Cohort will genetically correlate with related (albeit distinct) phenotypes from external GWAS. Specifically, negative affect will positively correlate with depression/anxiety and negatively correlate with wellbeing (reverse direction for positive affect), and higher cognitive performance on all domains will correlate with GCA; (iii) there will be evidence of bidirectional causal relationships between mental health and cognitive phenotypes. Figure 1 presents study objectives and design.

## Methods and materials

### Description of the lifelines cohort

Lifelines is a multi-disciplinary prospective population-based cohort study examining in a unique three-generation design the health and health-related behaviours of 167,729 persons living in the north of the Netherlands^26^. It employs a broad range of investigative procedures in assessing the biomedical, socio-demographic, behavioural, physical and psychological factors which contribute to the health and disease of the general population, with a special focus on multi-morbidity and complex genetics^26^. For more detail, see^26^ and Supplementary Methods and the Lifelines Website (www.lifelines-biobank.com). In this study, we included participants ≥ 18 years who were genotyped on a GWAS array, and we excluded participants who have conditions with a significant cognitive sequelae: Alzheimer’s disease, other dementia, epilepsy, multiple sclerosis, Parkinson’s disease, and stroke.

### Assessment of positive and negative affect

The Positive and Negative Affect Schedule (PANAS) was used. PANAS assesses positive and negative affect using two separate sub-scales, each containing 10 items (example items: excited, upset, nervous)^1,27^. In Lifelines, participants rate the extent to which they experienced each item during the last four weeks on a five-point scale (ranging from ‘not at all’ to ‘extremely’). The outcome is the summed score on each subscale, which ranges from 10–50 (higher values reflect higher positive or negative affect). The scales have high internal consistency (*a* = 0.87) and moderate test–retest reliability over an 8-week period (positive *r* = 0.58; negative *r* = 0.48)^1^.

### Assessment of cognitive performance

The Ruff Figural Fluency Test (RFFT) was used, which is a valid and reliable measure of executive functioning^28^. The task consists of five parts, each containing 35 identical five-dot patterns. Participants draw as many unique designs as possible within one minute by connecting dots in different patterns^29^. The primary outcome is the total number of unique designs.

Four tasks from the CogState Test Battery were administered in Lifelines, these tasks are designed to tap into specific cognitive domains: visual learning and memory (one card learning task), reaction time/attention (identification task and detection task), and working memory (one-back task). As the detection and identification task both assess reaction time, we include only the identification task in this study which has a larger sample size. We used the recommended primary outcomes for all tasks. All outcomes were transformed (reaction time [ms] on the identification task was log10 transformed, and accuracy on the one-back and one card learning task were arcsine transformed) in line with recommendations from Cogstate who developed these tasks (https://www.cogstate.com/digital-cognitive-assessment/). On both memory tasks, higher values reflect better memory; on the reaction time task higher values reflect poorer performance (i.e., slower reaction time). See Supplementary Methods for further information on dataset preparation.

### Genetics data and quality control

Genotyping was conducted in three subsets of the Lifelines cohort, each using a different chip array: (i) Illumina CytoSNP-12 Bead Chip v2 array (N = 17,033), (ii) Infinium Global Screening Array (GSA) Beadchip-24 v1.0 (N = 38,030), (iii) FinnGen Thermo Fisher Axiom® custom array (Affymetrix; N = 29,166). See Supplementary for quality control (QC) and imputation procedures conducted by Lifelines. Following Lifelines QCs, 73,086 participants were potentially eligible for this study (CytoSNP N = 14,942; GSA N = 31,810; Affymetrix N = 26,334). We applied additional QCs, removing: (i) duplicates (individuals genotyped on > 1 chip) and 1st-degree relatives between chips, (ii) non-European individuals, and (iii) genetic outliers, see Supplementary Figure S1. As we conducted GWAS within each Lifelines subset (i.e., we conducted three GWAS/phenotype which were subsequently meta-analysed), we also removed 1st-degree relatives between subsets because whilst REGENIE adjusts for relatedness *within* each subset, it will not account for relatedness *between* subsets. Consequently, to reduce the risk of relatedness impacting the results, these individuals were removed. Following additional QCs, 58,713 participants with genetics data were included in this study (CytoSNP N = 7632; GSA N = 24,975; Affymetrix N = 26,106).

### GWAS summary statistics for related mental health and cognitive phenotypes

To examine genetic overlap and potential causal relationships between affect and cognitive performance, we also used summary statistics from large publicly available GWAS of depression (N Cases = 294,322; N Controls = 741,438)^30^, anxiety (N Cases = 7,016; N Controls = 14,745)^31^, wellbeing (N = 2,370,390)^32^, general cognitive ability (GCA; N = 373,617)^33^, and brain volume (N = 47,316)^34^. For brain volume, some data includes head circumference as a proxy for brain volume (N = 18,881 of the total 47,316 participants). See Supplementary Methods and Table S1-S2 for details on these GWAS and how to access the summary statistics.

### Statistical analyses

#### GWAS of negative affect, positive affect, and cognitive performance in lifelines

We conducted GWAS (negative affect score, positive affect score, and performance on each cognitive task) in each genotyped subset separately using REGENIE, which accounts for relatedness within each subset^35^. Prior to conducting GWAS, we standardised all outcomes in the full phenotype file (mean = 0; SD = 1) and used PLINK^36^ to clean the genetics data for REGENIE Step 1. In REGENIE step 1, we used directly genotyped data and we included variants that met the following criteria: variant call rate (≥ 95%), Hardy–Weinberg equilibrium (1e-6), minor allele count (100), minor allele frequency (0.01), and restricted to individuals with low missingness (call rate ≥ 95%). For REGENIE step 2, we used imputed data. In each GWAS, covariates included age, sex, and top 10 genetic principal components. We excluded poorly imputed variants (INFO score < 0.8). For each outcome, we meta-analysed GWAS across the three subsets using STDERR model in METAL and applied genomic corrections^37^. GWAS quality was inspected using GWAS Inspector 1.6.4.0^38^. The standard *p*-value threshold of 5 × 10^–8^ was used to determine genome-wide significance (for suggestive significance: *p*-value  < 5 × 10^–6^).

### Tissue specificity of prioritised genes

We used gene and tissue mapping to explore associated variants in each GWAS. We used an online platform (FUMA) (https://fuma.ctglab.nl/) which integrates resources to annotate, prioritize, and visualise the summary statistics^39^. The SNP2GENE function was used to annotate SNPs and prioritise genes at each locus using positional mapping. Using these prioritised genes, we used GENE2FUNC to investigate tissue specificity of differentially expressed gene (DEG) sets using GTEx v8 data on 54 non-diseased tissue types (N ≤ 838 adults) (www.gtexportal.org/home/aboutAdultGtex). For further details, see Supplementary Methods.

#### Genetic correlations

Linkage Disequilibrium Score Regression (LDSR)^15,16^ was used to estimate genetic correlations between mental health and cognitive phenotypes. This included phenotypes from Lifelines (positive and negative affect, cognitive performance), and phenotypes from large external GWAS (depression, anxiety, wellbeing, GCA, brain volume). We used precomputed LD scores calculated using 1000G European data. As low imputation quality may confound LDSR we filtered to HapMap3 panel SNPs which tend to be well-imputed in most studies^15,16^. If GWAS did not have a sample size column for SNPs, we assumed the same sample size for all SNPs. We checked all phenotypes had heritability z-scores > 4 in line with standard procedure, all GWAS met this criterion except anxiety (Table S26)^15,16^. For LDSR cleaning filters applied, see^15,16^.

### Bidirectional Mendelian randomization

MR can overcome issues of confounding and reverse causality present in traditional observational studies, enabling causal inferences^17^. However, the validity of causal inferences drawn from MR relies on three key assumptions being met: (i) genetic variant(s) are robustly associated with the exposure, (ii) there is no confounding of the genetic variants(s) and the outcome, (iii) the genetic variant(s) are independent of the outcome given the exposure^21^. In the context of the traits being examined in this paper, horizontal pleiotropy (correlated and uncorrelated) and population-level confounding could violate MR assumptions. To address issues of horizontal pleiotropy, we employed several standard MR methods designed to deal with this (MR Egger, weighted median, weighted mode), and additionally applied a more recently developed method in the field designed to tackle both correlated and uncorrelated horizontal pleiotropy (MR CAUSE)^40–43^. Although not employed here, other MR methods have also been developed to detect and account for horizontal pleiotropy such as MR-PRESSO^44^. Additionally, we conducted MR analyses using within-sibship GWAS of similar traits where available. An additional assumption of two-sample MR (used here) is that samples come from the same underlying population but are non-overlapping^45^. However, recent work suggests that sample overlap may not bias MR results as much as previously thought, unless the instruments are not strongly associated with the exposure^21,46^.

MR was performed to investigate the potential causal effect of mental health phenotypes on cognition, and vice versa. This was done in R v4.2.2 using *TwoSampleMR*^47^ and *CAUSE* v1.2.0.0335^43^.

For standard MR methods, we applied the following criteria to identify genetic instruments: (i) *p* < 5 × 10^–8^ (if not available, a lenient *p* < 5 × 10^–6^ threshold was used) (ii) independent (r^2^ < 0.01, kb = 1000; based on European clustering in the 1000 genomes reference panel using *ld_clump()* in the *ieugwasr* package)^48^, (iii) minor allele frequency (MAF) ≥ 1%. For the depression GWAS, we confirmed that no variants had MAF < 1% in either cases or controls, so this filter was not applied for this phenotype. Any SNPs not available in the outcome GWAS were excluded. For primary analyses, we used the Inverse-Variance Weighted (IVW) method (> 1 SNP available) or Wald ratio (if only 1 SNP was available). Where multiple SNPs were available, we conducted sensitivity analyses using different MR methods which have different assumptions regarding the validity of the genetic instruments: MR-Egger^40^ weighted median^41^ weighted mode^42^. It is important to note that the IVW method which is often used as the primary method in MR analyses assumes that all genetic variants used as instruments are valid^41^. This is arguably an unrealistic assumption for many studies, including this study (e.g., due to potential horizontal pleiotropy). Consequently, we interpret our results in terms of consistency across multiple MR methods, which have varying assumptions regarding the validity of the genetic instruments.

We also applied Steiger filtering to assess whether genetic variants have stronger associations with the exposure than the outcome^47^. Given the possibility of correlated pleiotropy, we additionally applied causal analysis using summary effect estimates (CAUSE) which is designed to account for correlated and uncorrelated pleiotropy^43^. For MR CAUSE, we included genome-wide variants from the processed GWAS summary statistics and pruned variants using default criteria (r^2^ = 0.01, *p*-value  = 0.001) based on the European 1000 genomes reference panel. Given the possibility of population-level confounding (e.g., dynastic effects), we also conducted within-sibship MR using the MR-base platform^49^ and publicly available within-sibship GWAS on wellbeing (https://opengwas.io/datasets/ieu-b-4851) depressive symptoms (https://opengwas.io/datasets/ieu-b-4839), and cognitive function (https://opengwas.io/datasets/ieu-b-4837)^50^; see Supplementary methods for more detail. We also checked for heterogeneity (Cochran’s Q-statistic) and pleiotropy (Egger intercept). For further details on assumptions of different MR methods used here, see Supplementary methods.

In our primary analysis, we included phenotypes which have well-powered GWAS as exposures (≥ 75 SNPs after Steiger filtering: depression, wellbeing, GCA). In secondary analysis, we included phenotypes which have less well-powered GWAS as exposures (1 SNP or lenient *p*-value criteria: anxiety, negative affect, positive affect) to investigate whether similar patterns of effects are observed for these phenotypes.

## Results

### GWAS of negative affect, positive affect, and cognitive performance in the lifelines cohort

No SNPs met genome-wide significance threshold (*p* < 5 × 10^–8^), except one SNP for reaction time (rs2920287, β = 0.29, SE = 0.05, MAF = 0.04, *p* = 1.907e-08, N = 4,609). Visual inspection of the locus zoom plot of the region around rs2920287 shows many nearby genes, with *PSCA* being in closest proximity (Figure S4). Follow-up analyses of this SNP using the GWAS Catalog^51^ did not show associations with any traits at the time of the analysis. Using LDproxy^52^, SNPs in high LD with this SNP (r^2^ ≥ 0.6 within ± 1Mb) in CEU population (rs76679208, rs2280877, rs78969819) also did not show associations with any traits in the GWAS Catalog at the time of the analysis. Lowering the threshold to *p* < 5 × 10^–6^ yielded associated SNPs for all phenotypes (Table 1). For Manhattan plots, see Supplementary Figures S2-S3. For SNP-based heritability estimates calculated using LD score regression, see Supplementary Table S26. However, it is important to note that these estimates may be biased (downward) due to genomic corrections applied to the Lifelines GWAS. This means that SNP-based heritability estimates reported here may be underestimated. For Lifelines GWAS, SNP-based heritability estimates (range: from 0.07 to 0.11) and lambda GC (range: 1.04 to 1.07).

**Table 1:** GWAS results of positive affect, negative affect, and cognitive performance in the lifelines cohort.

Phenotype	Max N	Hits (*p* < 5e-08)	Hits Clumped (1000kb, r^2^ = 0.01)	Hits (*p* < 5e-06)	Hits Clumped (1000kb, r^2^ = 0.01)
Positive affect	57,946	0	0	67	15
Negative affect	57,946	0	0	73	20
Executive function	36,563	0	0	108	12
Visual learning and memory	36,783	0	0	139	12
Reaction time	35,729	1	1	176	19
Working memory	36,349	0	0	56	11

Note: Variants with MAF < 0.01 excluded.

#### Tissue specificity of prioritised genes

Given the limited number of SNPs meeting genome-wide significance, we used a lenient *p*-value threshold (*p* < 5 × 10^–6^) to identify SNPs for follow-up analysis in FUMA. As this is a more lenient threshold, these tissue enrichment analyses are exploratory and require further replication. For all phenotypes, prioritised genes were upregulated across multiple tissue types, particularly brain tissue (see Supplementary Figures S5-S6 and Tables S18-S23). Following FUMAs default Bonferroni corrections for multiple comparisons, there was evidence of upregulation in brain tissue for positive affect (substantia nigra) and visual learning and memory (cerebellum and cerebellar hemispheres) compared to other tissue types. For visualisation of tissue specificity of prioritised genes, see Supplementary Figures S5-S6.

### Genetic correlations between affect and mental health phenotypes

#### Negative affect is genetically correlated with mental health phenotypes

Negative affect score was genetically correlated with depression (*r*_g_ = 0.51 [SE = 0.05], *p* = 2.80E-28), wellbeing (*r*_g_ = − 0.71 [SE = 0.05], *p* = 8.65E-41), and anxiety (*r*_g_ = 0.70 [SE = 0.18], *p* = 2E-04). Positive affect showed weaker genetic correlations with depression (*r*_g_ = − 0.11 [SE = 0.04], *p* = 0.005) and wellbeing (*r*_g_ = 0.30 [SE = 0.05], *p* = 2.38E-09); and little evidence with anxiety (*r*_g_ = − 0.16 [SE = 0.17], *p* = 0.33).

#### Cognitive domains are genetically correlated with GCA

Cognitive performance on Lifelines tasks were genetically correlated with the largest GWAS (at the time of analysis) for GCA: executive function (*r*_g_ = 0.66 [SE = 0.07], *p* = 1.39E-23), visual learning and memory (*r*_g_ = 0.54 [SE = 0.05], *p* = 6.90E-29), working memory (*r*_g_ = 0.53 [SE = 0.06], *p* = 1.23E-18), and reaction time (*r*_g_ = − 0.39 [SE = 0.06], *p* = 1.48E-12).

#### Compared to positive affect, negative affect has a stronger genetic correlation with GCA

There was a weak negative genetic correlation between positive and negative affect scores (*r*_g_ = − 0.18 [SE = 0.08], *p* = 0.016). There was stronger evidence of genetic correlations between GCA and negative affect (*r*_g_ = − 0.19 [SE = 0.04], *p* = 7.56E-06) compared to GCA and positive affect (*r*_g_ = − 0.06 [SE = 0.04], *p* = 0.18).

**Fig. 2:**
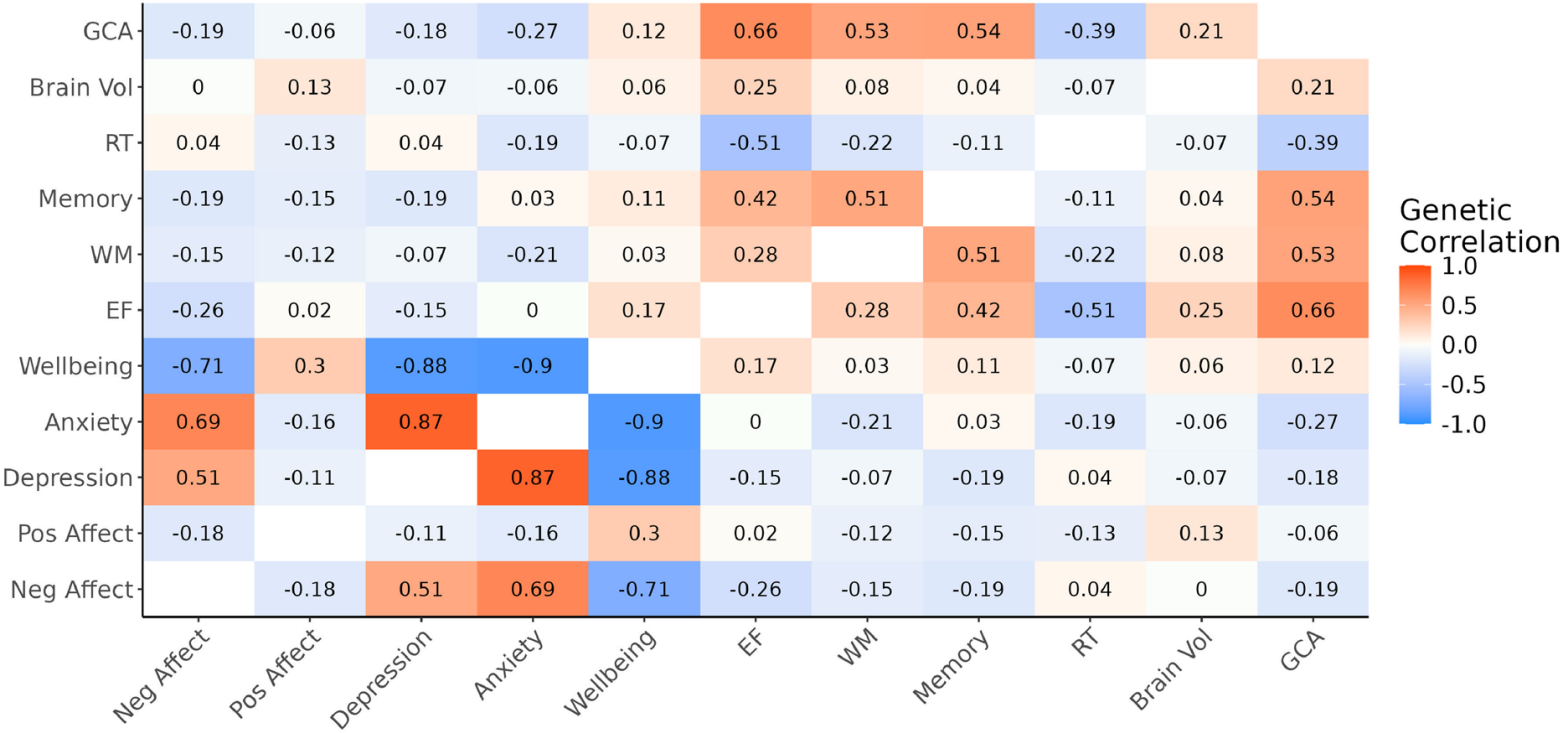
Genetic correlations between Lifelines phenotypes (negative affect, positive affect, cognitive performance) and external phenotypes (depression, anxiety, wellbeing, GCA, brain volume) using LDSR. Please note: all genetic correlation results are presented regardless of p-value criteria. GCA = General Cognitive Ability; Brain Vol = Brain Volume; RT = Reaction Time; Memory = Visual Learning and Memory; WM = Working Memory; EF = Executive Functioning; Pos Affect = Positive Affect (PANAS); Neg Affect = Negative Affect (PANAS).

For all results, see Fig. 2 and Supplementary Table S3.

### Potential causal effects: results from Mendelian randomization analyses

#### Effect of mental health phenotypes on cognition

**Fig. 3:**
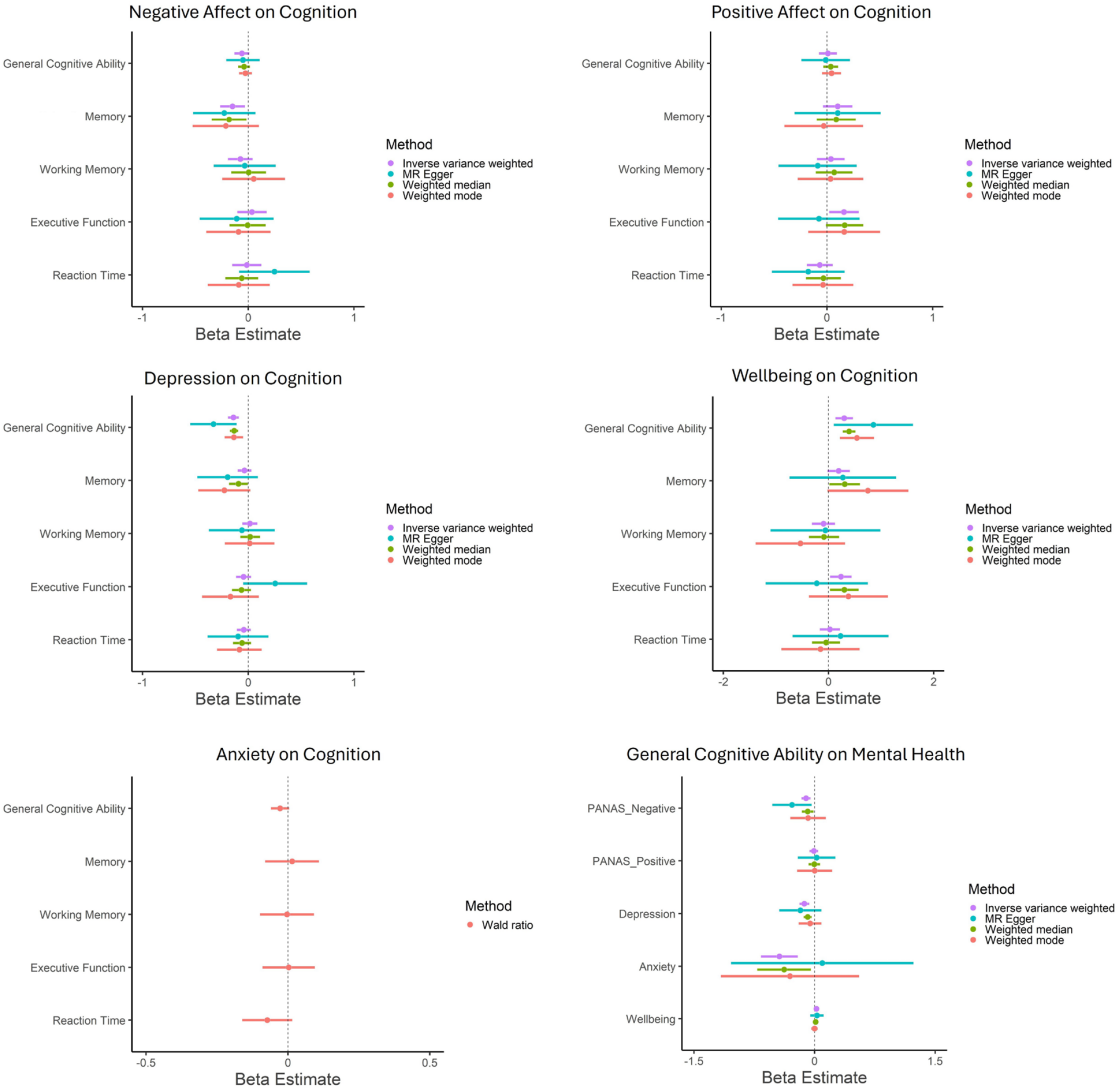
Mendelian randomization analyses testing evidence of potential causality between mental health and cognition phenotypes. Note: Bars reflect 95% CIs. Axes differ to ensure inclusion of CIs for all analyses. For binary phenotypes (anxiety, depression), beta reflects log(OR).

**Fig. 4:**
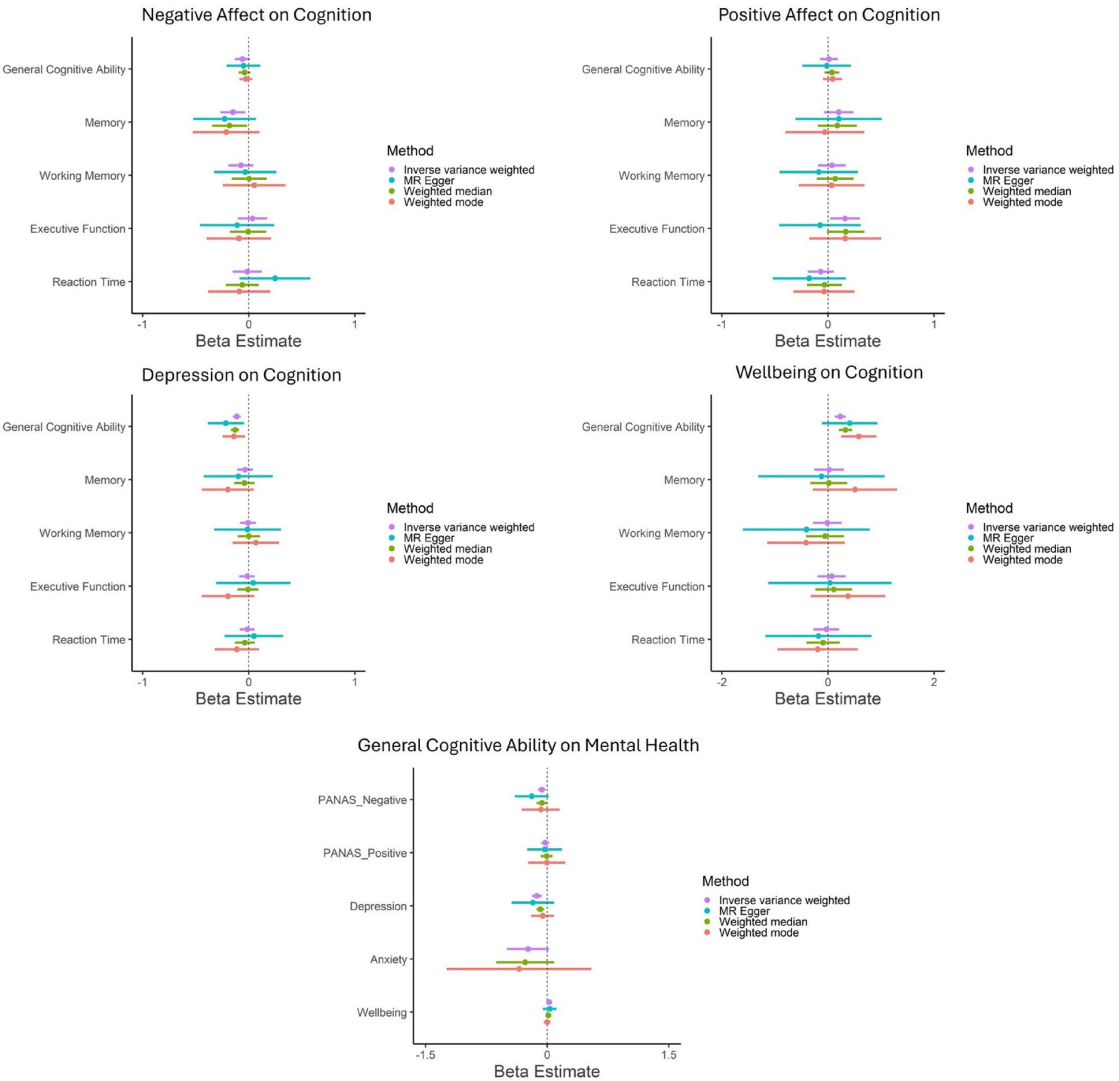
Steiger filtered Mendelian randomization analyses testing evidence of potential causality between mental health and cognition phenotypes. Note: Bars reflect 95% CIs. Axes differ to increase visibility of results in different analyses. Please note: The MR-Egger estimate for general cognitive ability on anxiety is not shown because its confidence interval is extremely wide and extends far beyond the plot limits, making it visually uninformative. For full results, please see Supplementary Table S5. For binary phenotypes (anxiety, depression), beta reflects log(OR).

In primary analyses, across all MR methods there was consistent evidence for a potential causal effect of depression on lower GCA (IVW estimate: − 0.14 [95% CI = − 0.19 to − 0.09], *p* = 6E-08, *Benjamini–Hochberg p* = *2E-06, Bonferroni p* = *2E-06*) and of wellbeing on higher GCA (IVW estimate: 0.30 [95% CI = 0.13 to 0.46], *p* = 4E-04, *Benjamini–Hochberg p* = *2E-03, Bonferroni p* = *1E-02)* (Fig. 3). These effects remained after applying Steiger filtering (Fig. 4; Supplementary Tables S6-S9) and were supported by MR CAUSE (Supplementary Table S24-S25). There was also evidence for a potential causal effect of wellbeing on better executive functioning (IVW estimate: 0.24 [95% CI = 0.03 to 0.44], p = 0.024, *Benjamini–Hochberg p* = *8E-02, Bonferroni p* = *7E-01*), and weak evidence of an effect on memory (IVW estimate: 0.19 [95% CI = − 0.02 to 0.41], p = 0.076, *Benjamini–Hochberg p* = *2E-01, Bonferroni p* = *1E* + *00*). However, these effects were not consistent across MR methods, and were not supported following Steiger filtering and correction for multiple comparisons (Fig. 4; Supplementary Tables S6-S9).

In secondary analyses, there was evidence for a potential causal effect of one SD increase in negative affect on poorer memory (IVW estimate: − 0.15 [95% CI = − 0.27 to − 0.03], *p* = 0.012, *Benjamini–Hochberg p* = *5E-02, Bonferroni p* = *4E-01*), and of one SD increase in positive affect on better executive functioning (IVW estimate: 0.16 [95% CI = 0.02 to 0.30], *p* = 0.024, *Benjamini–Hochberg p* = *8E-02, Bonferroni p* = *7E-01*). However, these effects were not consistent across MR methods and there was limited evidence after correction for multiple comparisons, see Figs. 4 and Supplementary Tables S10-S13.

For other mental health and cognitive phenotypes, there was little evidence of causality based on IVW estimates (Figs. 4; Supplementary Tables S6-S14). Confidence intervals (CIs) using within-sibship GWAS were very large with imprecise estimates (Supplementary Figure S16).

There was evidence of heterogeneity, with the strongest evidence in analyses of depression and wellbeing on GCA, see Supplementary Table S15. There was little evidence of horizontal pleiotropy (based on Egger Intercept; *p*s ≥ 0.14), except for weak evidence in MR analyses of depression on GCA (*p* = 0.084) and executive function (*p* = 0.048); see Supplementary Table S16.

#### Effect of GCA on mental health phenotypes

There was evidence for a potential causal effect of higher GCA on lower negative affect (IVW estimate: − 0.11 [95% CI = − 0.16 to − 0.05], p = 2E-04, *Benjamini–Hochberg p* = *2E-03, Bonferroni p* = *6E-03*), reduced risk of depression (OR: 0.88 [95% CI = 0.83 to 0.93], *p* = 3E-05, *Benjamini–Hochberg p* = *5E-04, Bonferroni p* = *1E-03*), and anxiety (OR: 0.64 [95% CI = 0.51 to 0.81], *p* = 2E-04, *Benjamini–Hochberg p* = *2E-03, Bonferroni p* = *5E-03*), and higher wellbeing (IVW estimate: 0.02 [95% CI = 0.01 to 0.04], *p* = 0.011, *Benjamini–Hochberg p* = *5E-02, Bonferroni p* = *3E-01*) (Fig. 3; Supplementary Table S4). However, effects were not consistent across all MR methods: there was more consistent evidence for GCA on negative affect (3/4 methods provide evidence supporting causality) compared to depression (2/4), anxiety (2/4), and wellbeing (1/4).

Following Steiger filtering, there was still evidence for a potential causal effect of higher GCA on lower negative affect (IVW estimate: − 0.07 [95% CI = − 0.11 to − 0.02], *p* = 0.007) but weaker evidence on reduced risk of anxiety (IVW estimate: − 0.24 [95% CI = − 0.50 to 0.02], *p* = 0.072), although CIs were large (see Fig. 4 and Supplementary Table S5). For GCA on depression and wellbeing, results were unaltered as no variants were removed in Steiger filtering. MR CAUSE support that the data are consistent with a potential causal effect of GCA on negative affect, depression, and wellbeing (See Supplementary Tables S24-S25). Although MR CAUSE suggests the data for GCA on anxiety fit the causal model better than the null or sharing model, this did not meet conventional *p*-value criteria (*p* > 0.05). In within-sibship MR, CIs were very large with imprecise estimates (Supplementary Figure S16).

There was strong evidence of heterogeneity in MR analyses testing effects of GCA on mental health phenotypes, except for GCA on anxiety (*p* = 0.34); see Supplementary Table S15. There was little evidence of horizontal pleiotropy based on MR Egger Intercept (*p*s ≥ 0.15) (Supplementary Table S16).

## Discussion

We conducted GWAS on negative and positive affect using a well-validated scale (PANAS) and four cognitive domains (executive function, working memory, visual learning and memory, reaction time) in the Lifelines Cohort. We identified one SNP that reached genome-wide significance (*p* < 5 × 10^–8^) for reaction time, and many independent SNPs with suggestive associations (*p* < 5 × 10^–6^) for other phenotypes. For most phenotypes, gene mapping shows that SNPs with suggestive associations have higher gene expression in brain tissue compared to other tissues. However, this only met Bonferroni-corrected *p*-value threshold criteria for positive affect and visual learning and memory, and as we used suggestive associations (due to the limited number of SNPs meeting genome-wide significance) these results should be considered as exploratory. Negative affect is genetically correlated with, yet distinct from, mental health phenotypes (depression *r*_g_ = 0.51; anxiety *r*_g_ = 0.70; wellbeing *r*_g_ = − 0.71) and cognitive domains are genetically correlated with GCA and brain volume (*r*_g_ ≤ 0.66). Genetic correlations suggest positive and negative affect are dissociable constructs (*r*_g_ = − 0.18); with negative affect having higher genetic overlap with GCA (*r*_g_ = − 0.19 vs − 0.06). MR results support potential causal effects of: (i) higher GCA on reduced negative affect, reduced depression and anxiety, and higher wellbeing, but little impact on positive affect; (ii) depression and lower wellbeing on reduced GCA. These results suggest that interventions indirectly influencing GCA may be valid targets for reducing negative affect, but not for increasing positive affect; and interventions indirectly influencing depression and wellbeing may be valid targets for GCA.

### Genetic correlations between affect and cognition

Genetic correlation analyses reveal that whilst there is moderate genetic overlap between negative affect and mental health phenotypes (depression *r*_g_ = 0.51; anxiety *r*_g_ = 0.70; wellbeing *r*_g_ = − 0.71), and between cognitive domains and cognition-related phenotypes (GCA, brain volume; *r*_g_ ≤ 0.66), they are not interchangeable but instead have partly distinct genetic components. Additionally, consistent with previous studies, we found evidence of a pleiotropic genetic architecture between cognitive and mental health phenotypes^18–20^. Specifically, genetic correlations between major depression and various cognitive measures in the Lifelines cohort (*r*_*g*_ range: − 0.07 to − 0.19, excluding reaction time for which higher values indicate poorer performance; *r*_*g*_= 0.04) are of a similar magnitude and direction to those reported by Hagenaars et al.^18^ using cognitive tasks from UK Biobank (*r*_*g*_ range: − 0.11 to − 0.25); whereby higher genetic liability to depression is associated with poorer cognition. Second, negative and positive affect are genetically dissociable constructs as opposed to being opposite ends of the same spectrum (*r*_g_ = − 0.18). Third, compared to positive affect, negative affect has higher genetic overlap with GCA (*r*_g_ = − 0.19 vs − 0.06) which could indicate that negative affect has a higher shared neural basis with GCA than positive affect and/or GCA and negative affect may exhibit causal relationships (as suggested by our MR results).

### Potential causality between mental health and cognitive phenotypes

Across MR methods, we found evidence of potential causal effects of higher GCA on many mental health outcomes (reduced negative affect, reduced risk of depression and anxiety, increased wellbeing). There was also evidence of potential causal effects of depression and wellbeing on GCA. Whilst we did not observe evidence for negative affect and anxiety on GCA, given the consistent direction of effect, this may be due to these GWAS being smaller and having decreased statistical power when used as exposures. Our findings complement recent studies using MR and complementary designs (e.g., within-sibship analyses) reporting evidence of causality between mental health phenotypes (depression, wellbeing, and/or anxiety) and constructs related to cognition (e.g., educational attainment^53^, education duration ^54^).

However, few studies have directly tested causality between mental health phenotypes and *cognition*. Using MR analyses in the ALSPAC cohort, Suddell et al.^12^ tested potential causality between mental health conditions (depression, anxiety) and specific cognitive domains (response inhibition, working memory, emotion recognition) but reported that estimates were imprecise, likely due to limited statistical power^12^. Marchi et al.^55^ used multivariable MR to test potential causal effects of GCA and poverty on several mental health conditions (including depression and anxiety)^55^. After adjusting for poverty, they found evidence that higher GCA may causally reduce risk of depression and anxiety. Through inclusion of additional GWAS (negative affect, positive affect, larger depression GWAS), our findings lend support to this, and additionally suggest that: (i) higher GCA may also cause lower negative affect (but not influence positive affect), (ii) bidirectional effects (i.e., higher risk of depression and lower wellbeing may also cause poorer GCA). Taken together, these MR findings suggest potential bidirectional causal relationships between mental health and cognition.

Despite the negative affect GWAS being much smaller than the depression GWAS (N = 57,946 versus N = 1,035,760), when used as an outcome, we found more consistent evidence across MR methods that GCA may play a causal role in negative affect. Speculatively, this may be because the negative affect GWAS consists of a more homogeneous phenotype which may increase statistical power and impact effect estimates. This highlights that GWAS of affect are valuable and may be informative beyond currently available GWAS on related yet distinct broad phenotypes (e.g., depression, wellbeing). Moreover, compared to personality trait GWAS which are thought to be less modifiable (e.g., neuroticism^56^), GWAS of affect may be more suited for downstream Mendelian randomization studies which aim to identify causal modifiable risk factors for affect-related phenotypes. Whilst we focused on positive and negative affect and specific cognitive domains, there is a need for GWAS on other transdiagnostic features (e.g., sleep disturbances, anhedonia, hot cognition^57^). It is likely that advances will also be gained by parsing heterogeneity using other approaches. For example, GWAS on depressed patients with specific characteristics (e.g., immune-metabolic depression^58^). Research focusing on improving the validity of subtypes within and across psychiatric conditions will be critical for advancing our understanding of these conditions^59.^

Whilst our results could indicate a bidirectional causal effect, other explanations (which violate MR assumptions) may account for these results including: (i) correlated pleiotropy and (ii) population-level confounding. Correlated pleiotropy occurs when genetic variants used in MR analyses affect both the exposure and outcome via a shared heritable factor^43^. A speculative example of how this could occur here is through genetic variants impacting brain-related processes (e.g., synaptic plasticity) which directly affect both cognition and mental health. Whilst we did include several MR methods which allow for some correlated pleiotropy (e.g., weighted median, weighted mode, MR CAUSE)^43^, these methods could still give biased estimates and false positives (leading to incorrect conclusions regarding causality) if the *majority* of instruments exhibit correlated pleiotropy. Overall, correlated pleiotropy remains a challenge for MR studies, particularly in relation to traits we focus on here. Another factor is population-level confounding (e.g., assortative mating, dynastic effects)^60^. Whilst we are interested in direct effects from GWAS (i.e., genetic variants effect on phenotypic variation), for many phenotypes, GWAS will also pick up indirect effects (e.g., dynastic effects: parental genotype affecting offspring phenotype via environmental factors). This violates MR assumptions and could lead to incorrect conclusions^60^. One approach proposed to overcome this is to use within-family GWAS^50,60^. Howe et al.^50^ found that GWAS estimates for some phenotypes, including cognitive function and depressive symptoms, were attenuated when using within-sibship GWAS compared to population-level GWAS^50^. To try to address this, we also conducted MR using within-sibship GWAS (depressive symptoms, wellbeing, and cognitive function) to test whether this impacted our findings. Unfortunately, confidence intervals (CIs) were very large with imprecise estimates (Supplementary Methods and Supplementary Figure S16). This is unsurprising given the much smaller sample size of within-sibship GWAS compared to population-level GWAS and highlights the need for larger within-family GWAS on complex phenotypes.

### Limitations

Limitations of this study must be considered when interpreting the results. First, smaller sample sizes for some GWAS resulted in a lack of genome-wide significant variants (*p* < 5 × 10^–8^) and/or larger CIs in MR analyses (affect, specific cognitive domains, anxiety). Findings with large CIs should therefore be considered inconclusive, and future studies using larger GWAS on these traits are required to more accurately estimate effects in genetic correlation and MR analyses. Consortia combining data from several large datasets are necessary to provide well-powered GWAS on these phenotypes; our GWAS in the Lifelines Cohort will provide a useful contribution to this endeavour. It is worth noting that newer GWAS for traits included in this paper are now available^61–63^ but were not used in this study as they were not available at the time of analysis. Additionally, genomic corrections applied to GWAS during meta-analysis using METAL may be overly conservative resulting in reduced statistical power to detect associations in our GWAS. Second, MR estimates the lifetime effect of an exposure (e.g., depression) on an outcome (e.g., GCA)^21^. Whilst this study is informative for understanding lifetime risk, it is unclear what time periods would be best to intervene on. This requires either a randomised controlled trial (RCT; which would be expensive and time consuming) or MR with large GWAS on exposures and outcomes at specific ages in the lifespan^64,65^. Third, there is sample overlap between some exposure-outcome GWAS used in MR analyses. Nevertheless, recent research suggests that sample overlap may not bias MR results as much as previously thought, unless the instruments are not strongly associated with the exposure^21,46^. In primary MR analyses, we used large exposure GWAS (that had ≥ 75 SNPs following Steiger filtering) to reduce the risk of weak instrument bias. However, secondary MR analyses investigating positive and negative affect on specific cognitive domains included substantial sample overlap (both GWAS were conducted the Lifelines Cohort) and included a lenient *p*-value threshold to identify SNPs associated with the exposure. Nevertheless, we did not observe evidence of effects in secondary analysis and therefore the potential for false positives is low. Fourth, cognitive performance is highly related to other socioeconomic phenotypes (e.g., education, socioeconomic status)^66^. Future studies testing independent effects and interactions between these phenotypes on mental health using other methods (e.g., multivariable MR) would be useful. Fifth, many GWAS use data from large population-based cohorts which are less representative of some populations (e.g., individuals from less affluent backgrounds), which may limit the generalizability of the findings. Sixth, for continuous variables (e.g., GCA), our study cannot shed light on whether relationships are nonlinear. As currently available nonlinear MR approaches have yielded implausible results^67^, there is a need for alternative methods to better characterise the shape of these relationships^68^. Seventh, we focus on a subset of phenotypes, future studies should expand this to provide insight into other mental health and cognitive phenotypes which may show different relationships (e.g., schizophrenia, hot cognition)^69^. Additionally, affect was measured at a single time-point using a four-week recall period. Versions of the PANAS that have shorter temporal instructions (e.g., past few weeks) are less stable over time than versions with longer temporal instructions (e.g., past year, in general)^1^. A single assessment fails to capture within-person variability in affect. Future studies should consider incorporating repeated measures of affect at (i) critical stages in the lifespan to examine potential sensitive periods and longitudinal within-person change^70,71^ and (ii) shorter time scales to better assess affect at a given time point (e.g., ecological momentary assessment). Finally, many GWAS on psychiatric conditions do not exclude people with comorbidities. For example, the depression GWAS includes UK Biobank which defines depression based on the following question: “Have you ever seen a general practitioner (or psychiatrist) for nerves, anxiety, tension, or depression?”^72^. This may result in many individuals with anxiety being characterised as having depression and makes it challenging to conduct subsequent analyses testing genetic overlap/causality between different conditions.

### Implications

Our GWAS of positive and negative affect and cognitive domains in the Lifelines Cohort provide valuable resources which may facilitate insights into the aetiology, comorbidity, and causal risk factors for these phenotypes. Whilst our MR results provide evidence of potential causal relationships between mental health phenotypes (negative affect, depression, anxiety, wellbeing) and GCA, future studies are needed to triangulate these findings with other study designs that have different strength/limitations to MR. If multiple lines of evidence support causality, this would suggest that strategies indirectly targeting poor mental health may prevent/treat cognitive dysfunction, and vice versa. However, it is also important to consider whether interventions indirectly targeting GCA could have a *strong enough* influence on elevated negative affect to be considered promising intervention targets. Further studies would be required to estimate the effect size of potential interventions. The effect size may be influenced by factors such as the specific intervention (e.g., interventions with reduced risk of ‘fade out’ over time may be needed) and population (e.g., individuals who are younger and at higher risk of cognitive challenges may benefit more), and therefore these factors must be carefully considered. Considering the broader literature^53,55^, policy changes targeting factors impacting GCA (e.g., education^73^) may be promising for reducing future mental health challenges.

Nevertheless, there is also evidence suggesting that cognitive impairments can persist in remitted depressed individuals^74^. This may appear to contrast the idea that treating depression may help to prevent/treat cognitive impairments. Speculatively, this could be because: (i) once depressive symptoms have decreased, cognitive impairments reduce but require a longer time to observe effects (RCTs may not have long enough follow-up lengths), (ii) reducing depression may help prevent cognitive impairments, but may not help treat them once they are already experienced, (iii) treating depression may improve cognition in a subset of people (not all individuals), and/or (iv) some symptoms of depression (but not all) when treated may improve cognition. There is a need for research testing these different theories to better understand the dynamic relationship between depression and cognition.

## Conclusions

In summary, we identified one SNP that reached genome-wide significance for reaction time, and many independent SNPs with suggestive associations for affect and specific cognitive domains. The genetic correlation and MR results provide evidence of modest genetic overlap between cognitive and mental health traits, and potential bidirectional causal relationships. The most robust evidence of a potential causal effect of GCA on mental health traits examined here was on negative affect. These findings suggest that interventions indirectly targeting GCA may be promising targets for negative affect (but not positive affect), and interventions for depression and wellbeing may be promising targets for GCA. However, given the challenges of applying MR to these traits, further studies using complementary research designs are warranted, particularly studies testing different theories we proposed in the discussion.

## Supplementary Information

Supplementary Information.

## Data Availability

Due to the sensitivity of the data involved, these data are published as a restricted dataset at the University of Bristol Research Data Repository data.bris, at 10.5523/bris.2uooem4mn8d062nyriuy7m5oyr. The metadata record published openly by the repository at this location clearly states how data can be accessed by bona fide researchers. Requests for access will be considered by the University of Bristol Research Data Service, who will assess the motives of potential data re-users before deciding to grant access to the data. No authentic request for access will be refused and re-users will not be charged for any part of this process.
